# Modifiable and non-modifiable risk factors affecting surgical failure after revision ACL reconstruction: a cohort study

**DOI:** 10.1186/s43019-024-00243-4

**Published:** 2024-11-25

**Authors:** Hyun-Soo Moon, Min Jung, Chong-Hyuk Choi, Kwangho Chung, Se-Han Jung, Junwoo Byun, Jin-Gyu Kim, Seungmin Lee, Sung-Hwan Kim

**Affiliations:** 1 Arthroscopy and Joint Research Institute, Yonsei University College of Medicine, Seoul, Republic of Korea; 2 Department of Orthopedic Surgery, Severance Hospital, Yonsei University College of Medicine, Seoul, Republic of Korea; 3 Department of Orthopedic Surgery, Yongin Severance Hospital, Yonsei University College of Medicine, Yongin, Republic of Korea; 4 Department of Orthopedic Surgery, Gangnam Severance Hospital, Yonsei University College of Medicine, Seoul, Republic of Korea

**Keywords:** Revision anterior cruciate ligament reconstruction, Surgical failure, Multiple revision surgery, Osteoarthritis, Femoral tunnel

## Abstract

**Background:**

Research on factors influencing the outcomes of revision anterior cruciate ligament (ACL) reconstruction is relatively scarce and mostly relies even on reports from a single group. Understanding the factors contributing to the failure of revision ACL reconstruction can provide valuable information for achieving better clinical outcomes and assist in patient counseling before surgery. Therefore, this study aimed to analyze the factors contributing to surgical failure after revision ACL reconstruction.

**Methods:**

The medical records of consecutive patients who underwent single-bundle revision ACL reconstruction using the transportal technique between 2010 and 2020 and had a minimum follow-up of 2 years were retrospectively reviewed. Eligible patients were classified into two groups on the basis of the presence of surgical failure during the follow-up period (group NF, patients who did not experience surgical failure; group F, patients who experienced surgical failure). In this study, surgical failure after revision ACL reconstruction was defined as meeting any of the following conditions during follow-up: the presence of graft re-tear confirmed by magnetic resonance imaging (MRI), anterior–posterior laxity graded ≥ 2, or rotational laxity graded ≥ 2. A comparative analysis was conducted on demographic data, as well as peri-, intra-, and postoperative data between the groups. Additionally, a regression analysis was performed to investigate factors influencing surgical failure after revision ACL reconstruction.

**Results:**

A total of 58 patients were included (group NF, 40 patients; group F, 18 patients). In between-group comparisons of demographic, peri-, and intra-operative data, group F exhibited a higher frequency of multiple revision surgeries (*P* = 0.001), increased preoperative osteoarthritis grade (*P* = 0.001), and shallower femoral tunnel depth (*P* = 0.002) compared with group NF. At the final follow-up, group F demonstrated relatively poor clinical outcomes, both subjectively and objectively. Multivariate regression analysis revealed that all variables that showed differences in the preceding comparisons were independent factors affecting surgical failure after revision ACL reconstruction.

**Conclusions:**

Surgical failure after revision ACL reconstruction can occur in a substantial number of patients, influenced by non-modifiable factors, such as cases corresponding to multiple revision surgery and preoperative osteoarthritis grade, and modifiable factors, such as femoral tunnel depth.

**Supplementary Information:**

The online version contains supplementary material available at 10.1186/s43019-024-00243-4.

## Background

With the increasing frequency of primary anterior cruciate ligament (ACL) reconstruction, there is a simultaneous rise in the occurrence of revision ACL reconstruction [1]. Despite ongoing improvements in surgical outcomes of primary ACL reconstruction owing to the accumulation of related knowledge and advancements in surgical techniques [2], instances of surgical failure necessitating revision ACL reconstruction persist [3, 4]. Indeed, the need for revision surgery was reported in up to 7.7% of patients who underwent primary ACL reconstruction [5, 6], with an estimated annual incidence of around 13,000 such procedures in the USA [7].

Nevertheless, the surgical outcomes of revision ACL reconstruction are known to be relatively inferior and unpredictable compared with primary ACL reconstruction [8–11]. Although it varies depending on the definition of surgical failure [12], it has been reported that surgical failure after revision ACL reconstruction can be more than 20% [3]. This may be attributed to inadequate addressing of factors contributing to surgical failure of the primary ACL reconstruction (e.g., non-anatomic tunnel placement, graft type, untreated meniscus, cartilage, or ligament pathologies, malalignment of the lower extremity, increased tibial slope, etc.) or may arise from technical challenges [1, 13–16]. In fact, revision ACL reconstruction requires consideration of numerous variables compared with primary surgery [17]. Additionally, factors beyond the control of the patient or surgeon, as well as those not yet identified, might influence relatively poor results. Despite the aforementioned conditions, research on factors influencing the outcomes of revision ACL reconstruction is relatively scarce and mostly relies on reports from a single group [14, 18, 19]. Understanding the factors contributing to the failure of revision ACL reconstruction can provide valuable information for achieving better clinical outcomes and assist in patient counseling before surgery.

Therefore, this study aimed to analyze the factors contributing to surgical failure after revision ACL reconstruction. It was hypothesized that there are potential modifiable factors influencing surgical failure after revision ACL reconstruction.

## Methods

**Fig. 1:**
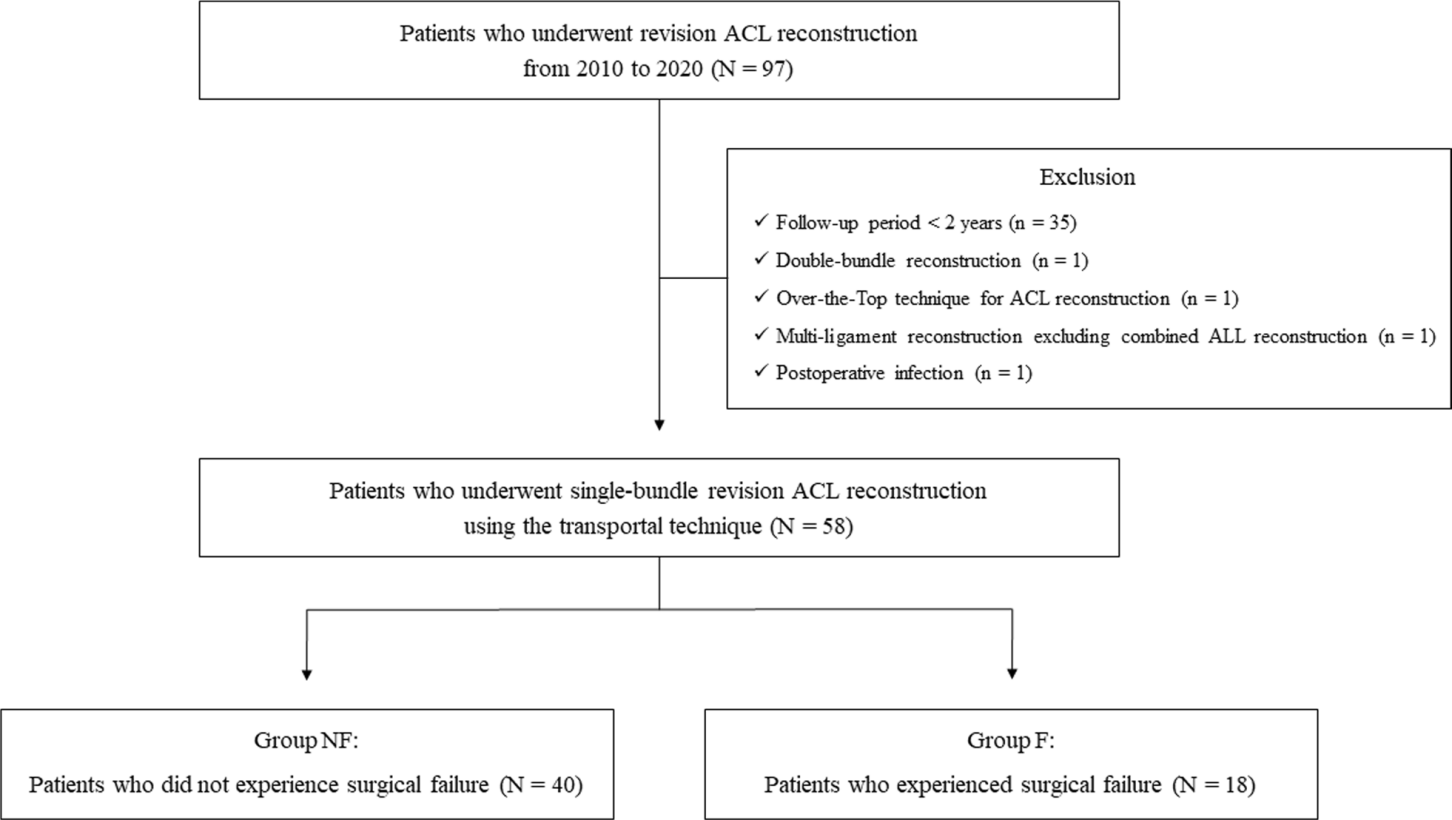
Patient flowchart

The current study obtained approval from our institution’s institutional review board, and the requirement for informed consent was waived owing to the retrospective nature of the research. The medical records of consecutive patients who underwent revision ACL reconstruction by two senior surgeons at a single institution from 2010 to 2020 were retrospectively reviewed. The inclusion criteria comprised patients who underwent single-bundle revision ACL reconstruction using the transportal (anteromedial portal) technique and had a minimum follow-up period of 2 years. To examine the effect of multiple revision surgery, cases where revision ACL reconstruction was performed more than once (e.g., re-revision surgery) were inclusively considered as instances of revision ACL reconstruction for analysis. Exclusion criteria were as follows: (1) a follow-up period of less than 2 years, (2) double-bundle reconstruction, (3) utilization of surgical techniques other than the transportal technique, (4) multiligament reconstruction (excluding combined anterolateral ligament reconstruction), and (5) postoperative infection. Subsequently, patients meeting the aforementioned inclusion criteria were classified into two groups on the basis of the presence of surgical failure during the follow-up period, including those without surgical failure (group NF) and those encompassing surgical failure (group F) (Fig. 1).

### Definition of surgical failure

Currently, there remains a lack of consensus on the definition of surgical failure after ACL reconstruction or revision ACL reconstruction. Depending on literature or authors, surgical failure may be defined simply as graft retear and could encompass functional insufficiency associated with ACL reconstruction [1, 20, 21]. In this study, by referencing prior research [3], surgical failure after revision ACL reconstruction was deemed to be present if any of the following conditions, directly associated with the outcomes of revision ACL reconstruction, were identified during the follow-up period: (1) graft re-tear confirmed by magnetic resonance imaging (MRI), (2) anterior–posterior (AP) laxity of the knee graded as 2 or 3 (on the basis of measurements using the KT-2000 arthrometer or manual Lachman test), and (3) rotational laxity of the knee graded as 2 or 3 (on the basis of measurements using the manual pivot-shift test). This criterion was adopted to encompass all possible conditions associated with unfavorable clinical outcomes after surgery.

### Surgical procedures and postoperative rehabilitation

The surgical technique and postoperative rehabilitation for revision ACL reconstruction were fundamentally executed in a manner identical to that of the primary surgery [22, 23], while also adhering to the principle of identifying and addressing the causes of failure in primary ACL reconstruction [17]. In most cases, single-bundle reconstruction using the transportal technique was employed. The choice of the ACL graft was determined on the basis of the type of graft used in the previous surgery and preoperative consultations with the patient. The fixation in the femoral tunnel of the graft was accomplished using a fixed-loop cortical suspension device (EndoButton CL; Smith & Nephew, Memphis, Tennessee, USA) for soft tissue grafts and a bioabsorbable interference screw for bone-patellar tendon-bone grafts. In the tibial tunnel, fixation was uniformly achieved with a bioabsorbable interference screw, with the inclusion of a cortical screw and washer for additional reinforcement in the case of soft tissue grafts. Consideration for a two-stage revision ACL reconstruction was given when tunnel widening exceeded approximately 15–16 mm [16].

### Patient evaluation

The evaluation of patients involved conducting clinical assessments, encompassing demographic data, functional scores, and knee laxity, along with intraoperative data and various radiographic parameters. Functional scores used for patient evaluation included International Knee Documentation Committee (IKDC) subjective, Lysholm, and Tegner activity scores [24, 25]. The assessment of knee laxity was conducted for both AP and rotational laxity. AP laxity of the knee was recorded using the KT-2000 arthrometer (MEDmetric, San Diego, California, USA) and the manual Lachman test [26]. Rotational laxity of the knee was evaluated through the manual pivot shift test and recorded as follows: grade 0 (absent = normal), grade 1 (glide = nearly normal), grade 2 (jump = abnormal), and grade 3 (transient lock = severely abnormal). The assessments of functional scores and knee laxity were based on records conducted independently of this study when patients visited outpatient clinics. In this study, the analysis was performed using records from the preoperative phase and the final follow-up. Additionally, clinical improvement beyond the minimal clinically important difference (MCID) was assessed for each functional score by examining the score differences between the two time points (16.7 for the IKDC subjective score, 8.9 for the Lysholm score, and 1 for the Tegner activity score) [27, 28].

The analysis of intraoperative data entailed the examination of the characteristics of the ACL graft and the state of the meniscus and cartilage on the basis of records at the time of surgery. The ACL grafts were evaluated for their diameter and specific type. The condition of the meniscus was classified for both the medial and lateral meniscus as functional, non-functional, or repaired [29]. A functional meniscus was defined as having no pathologic lesions or undergoing partial meniscectomy for conditions not affecting its functionality [29]. Non-functional meniscus was defined in cases where previous meniscectomy had been performed or in the presence of large irreparable meniscus lesions [29]. Cartilage was categorized for each compartment as intact or having a low-grade lesion, a high-grade lesion, or having undergone cartilage restoration procedures. In cases where cartilage lesions corresponded to ICRS grade 2 or below, they were identified as low-grade lesions, while lesions rated ICRS grade 3 or above were categorized as high-grade lesions. Furthermore, whether combined anterolateral ligament reconstruction was performed was assessed.

**Fig. 2:**
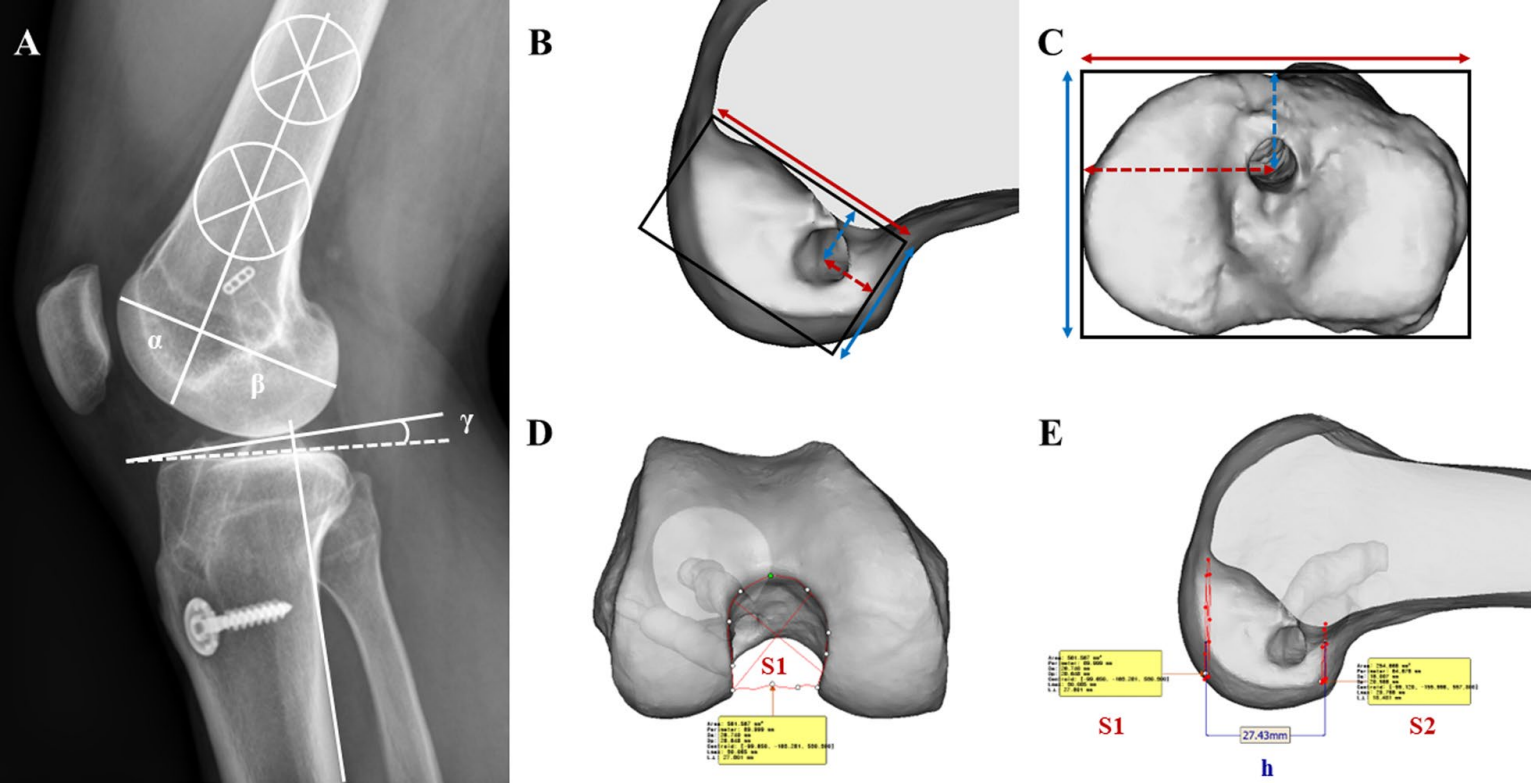
**A** Measurements of the lateral femoral condylar ratio (β/α + β × 100, %) and posterior tibial slope (γ, °). **B** Measurements of the ACL femoral tunnel position using a rectangular reference frame, indicating the tunnel height (dashed blue line/solid blue line × 100, %) and tunnel depth (dashed red line/solid red line × 100, %). **C** Measurement of the ACL tibial tunnel position with a rectangular reference frame, delineating the tunnel height (dashed blue line/solid blue line × 100, %) and tunnel width (dashed red line/solid red line × 100, %). **D**, **E** Femoral intercondylar notch volume was calculated with the following formula: volume (cm^3^) = h (S1 + S2 + √(S1S2))/3. In this equation, S1 and S2 denote the cross-sectional area of the intercondylar notch at the most distal and proximal levels of Blumensaat’s line, respectively, while h signifies the vertical distance between the two areas of the intercondylar notch

The radiographic evaluation involved the use of plain radiographs and computed tomography (CT) images. Assessment through plain radiographs involved evaluating the osteoarthritis grade in standing AP knee radiographs, measuring the posterior tibial slope and lateral femoral condyle ratio in lateral knee radiographs, and determining the hip-knee-ankle angle in full-length weight-bearing lower extremity AP radiographs (Fig. 2) [30, 31]. Additionally, the radiographic osteoarthritis grade was assessed at both the preoperative and final follow-up time points. Subsequently, postoperative CT images taken on the day of surgery were used to evaluate the position of the ACL tunnels in the femur and tibia, as well as the characteristics of the femoral intercondylar notch. The variables were assessed using a three-dimensionally (3D) reconstructed knee model. For this purpose, Digital Imaging and Communications in Medicine data from CT scans were imported into Mimics software (version 17; Materialize, Leuven, Belgium), facilitating the meticulous creation of 3D models of the femur and tibia. Initially, for evaluating ACL tunnel positions, femur and tibia models were rotated and aligned following the methodology of a prior study [32]. At these positions, the lateral wall of the intercondylar notch of the femur was employed as a rectangular reference frame to assess the femoral tunnel position (represented as height and depth), while the cortical outline of the proximal tibia served as a reference frame for evaluating the tibial tunnel position (represented as width and depth) (Fig. 2) [32]. The characteristics of the femoral intercondylar notch were also determined using a 3D femur model. Following the truncated-pyramid shape simulation method of a previous study [33], the cross-sectional area at the most distal and proximal positions of the intercondylar notch and the height of the intercondylar notch were measured (Fig. 2). Subsequently, these values were utilized to calculate the femoral intercondylar notch volume [33, 34]. An experienced orthopedic surgeon who had completed a fellowship in orthopedic surgery, blinded to patient information, conducted radiographic measurements twice over a 4-week interval to evaluate the measurement reliabilities. Continuous variables were analyzed by averaging two measurements. For categorical variables, any discrepancies between observers were resolved through discussion until a consensus was reached. In cases where discrepancies persisted, consultation with the senior author was sought for decision.

### Statistical analysis

All statistical analyses were performed using IBM SPSS Statistics for Windows (v26.0; Armonk, NY, USA). For the comparative analysis of continuous variables, Student’s *t*-test was used when a normal distribution was evident in each group, while the Mann–Whitney *U* test was implemented when the assumption of normal distribution was unmet. For categorical variables, either Pearson’s chi-square test or Fisher’s exact test was employed. Additionally, a multivariate logistic regression analysis was performed with variables that exhibited statistically significant differences in the comparisons between the two groups. The reliability of radiographic measurements was evaluated by calculating intraclass correlation coefficients (ICCs) with a confidence level set at 95%. The significance level was established at *P* < 0.05. Furthermore, a post hoc power for the regression analysis was calculated using G*POWER software (version 3.1.9.2; Heinrich Heine Universität, Düsseldorf) using the number of patients included in this study, with the significance level set at 5%.

## Results

A total of 58 patients were enrolled in this study, comprising 40 in group NF and 18 in group F. Within group F, surgical failure was attributed to various reasons, including six patients of graft retear, two patients of knee AP laxity ≥ grade 2, four patients of knee rotational laxity ≥ grade 2, and six patients with more than two reasons for failure. One patient in group NF and seven patients in group F underwent re-revision ACL reconstruction, with no instances of tertiary revision surgeries. While there were mostly no statistical differences in baseline demographic data between the groups, the number of patients corresponding to multiple revision ACL reconstruction were significantly higher in group F (*P* = 0.001) (Table 1). As multiple revision surgeries could independently influence the analysis results, this study conducted an additional comparison between groups, excluding patients who had undergone multiple revision surgeries to eliminate this potential influence. In the comparative analysis excluding patients undergoing multiple revision ACL reconstruction, no between-group differences were observed in baseline demographic data (Supplementary Table 1).

**Table 1:** Comparison of baseline demographic data

Variables^*a*^	Group NF (*N* = 40)	Group F (*N* = 18)	*P* Value
Age, years	27.8 ± 9.2	27.4 ± 10.1	0.717
Sex^*b*^			
Male/ Female	35/ 5	15/ 3	0.694
Affected side^*b*^			
Right/ Left	34/ 6	12/ 6	0.161
Body Mass Index, kg/m^2^	26.0 ± 3.6	24.8 ± 3.3	0.215
Postoperative CT scan^*bc*^			
Yes/ No	37/ 3	16/ 2	0.641
Postoperative MRI^*bd*^			
Yes/ No	27/ 13	12/ 6	> 0.999
Preinjury Tegner activity score	5.3 ± 1.8	5.1 ± 1.5	0.517
Follow-up duration, years	3.2 ± 1.4	3.4 ± 1.3	0.485
Multiple revision surgery^*b*^			
Yes/ No	1/ 39	7/ 11	0.001
Reason corresponding to surgical failure^*b*^			
Graft retear	NA	6	
Anteroposterior laxity ≥ grade 2	NA	2	
Rotational laxity ≥ grade 2	NA	4	
More than two reasons	NA	6	

*CT* computed tomography, *MRI* magnetic resonance imaging, *ACL* anterior cruciate ligament, *NA* not applicable

^*a*^The values are given as the mean and standard deviation, otherwise noted separately

^*b*^The values are given as number of patients

^*c*^Images taken immediately after surgery

^*d*^Images taken 1 year after surgery

In the realm of perioperative data, there were no observed differences between the two groups in preoperative functional scores and knee laxity. However, concerning preoperative radiographic variables, osteoarthritis grade was significantly higher in patients in group F (*P* = 0.001). Furthermore, a comparative assessment utilizing postoperative CT images revealed that the depth of the femoral tunnel in group F was relatively shallow compared with that in group NF (*P* = 0.002) (Table 2). Intraoperative data, encompassing graft diameter and type, meniscus and cartilage conditions, and the implementation of combined ALL reconstruction, showed no discernible differences between the two groups (Table 3). Similarly, in the comparison of peri- and intraoperative data excluding patients undergoing multiple revision ACL reconstruction, group F showed significantly higher preoperative osteoarthritis grade and shallower femoral tunnel depth, consistent with the earlier analysis (*P* = 0.003 and *P* = 0.007, respectively) (Supplementary Table 2 and 3). The 95% confidence intervals of the ICCs for radiographic parameters corresponding to continuous variables ranged from 0.91 to 0.987.

**Table 2:** Comparison of perioperative data

Variables^*a*^	Group NF (*N* = 40)	Group F (*N* = 18)	*P* value
Preoperative			
Functional scores			
IKDC subjective score	50.1 ± 15.4	57.2 ± 15.1	0.111
Lysholm score	60.6 ± 23.5	68.7 ± 21.8	0.223
Tegner activity score	1.9 ± 1.6	2.3 ± 1.2	0.182
Knee laxity			
SSD in anterior tibial translation, 134N	6.5 ± 2.8	7.1 ± 3.1	0.436
Lachman test, grade^*b*^			
0/ 1/ 2/ 3	0/ 10/ 21/ 9	1/ 2/ 10/ 5	0.337
Pivot-shift test, grade^*b*^			
0/ 1/ 2/ 3	1/ 8/ 18/ 12	0/ 5/ 4/ 9	0.287
Radiographic parameters			
Kellgren–Lawrence grade^*b*^			
0/ 1/ 2	27/ 11/ 2	4/ 7/ 7	0.001
Hip-knee-ankle angle, °	1.1 ± 3.4	0.4 ± 4.1	0.501
Posterior tibial slope, °	8.3 ± 2.8	8.8 ± 2.7	0.529
Lateral femoral condyle ratio, %	65.5 ± 2.7	66 ± 3.9	0.607
Postoperative			
Femoral tunnel position^*c*^			
Height, %	48.4 ± 7.8	45.2 ± 8.1	0.172
Depth, %	28.7 ± 5.3	34.2 ± 6.7	0.002
Tibial tunnel position^*c*^			
Depth, %	38 ± 4.4	40.3 ± 5.9	0.088
Width, %	44.8 ± 2.2	45.7 ± 1.3	0.137
Femoral intercondylar notch character^*c*^			
Volume, cm^3^	9.7 ± 1.9	9.7 ± 2.0	0.985
Distal base area, mm^2^	478.1 ± 74	470.4 ± 62	0.717
Proximal base area, mm^2^	244.2 ± 41.3	234.6 ± 44.1	0.451
Height, mm	27.3 ± 2.6	27.9 ± 2.8	0.448

*IKDC* International Knee Documentation Committee, *SSD* side-to-side difference

^*a*^The values are given as the mean and standard deviation, otherwise noted separately

^*b*^The values are given as number of patients

^*c*^For patients who underwent postoperative CT scan (37 in Group NF and 16 in Group F)

**Table 3:** Comparison of intraoperative data

Variables^*a*^	Group NF (*N* = 40)	Group F (*N* = 18)	*P* value
Graft diameter, mm^*b*^	9.2 ± 0.6	9.2 ± 1.0	0.694
Graft type			
Autograft/ allograft/ hybrid graft	5/ 32/ 3	5/ 12/ 1	0.362
Graft type (detailed)			
Hamstring autograft/ BTB autograft/ tibialis anterior allograft/ Achilles allograft/ hybrid graft	2/ 3/ 31/ 1/ 3	3/ 2/ 10/ 2/ 1	0.226
Associated intra-articular lesion			
Yes/ No	23/ 17	11/ 7	> 0.999
Medial meniscus			
Functional/ non-functional/ repair	22/ 5/ 13	10/ 4/ 4	0.583
Lateral meniscus			
Functional/ non-functional/ repair	34/ 0/ 6	16/ 0/ 2	> 0.999
Tibiofemoral joint cartilage			
Intact or low-grade lesion/ high-grade lesion/ restoration procedure	33/ 2/ 5	16/ 1/ 1	0.842
Patellofemoral joint cartilage			
Intact or low-grade lesion/ high-grade lesion/ restoration procedure	38/ 0/ 2	15/ 1/ 2	0.239
Combined ALL reconstruction			
Yes/ No	8/ 32	5/ 13	0.516

*ALL* anterolateral ligament, *BTB* bone-patellar tendon-bone

^*a*^The values are given as number of patients

^*b*^The values are given as the mean and standard deviation

In the between-group comparisons at the final follow-up, there were no differences in functional scores, but group F exhibited a significantly lower frequency of clinical improvement beyond the MCID value for the IKDC subjective score (*P* = 0.044). Both AP and rotational laxities of the knee were also significantly poor in group F (*P* < 0.001 for side-to-side difference in anterior tibial translation, *P* < 0.001 for the Lachman test, and *P* < 0.001 for the pivot-shift test). Furthermore, the radiographic osteoarthritis grade at the final follow-up was also notably higher in group F (*P* < 0.001) (Table 4). The between-group comparison of postoperative data, excluding patients undergoing multiple revision ACL reconstruction, also exhibited a trend similar to the aforementioned findings (Supplementary Table 4).

**Table 4:** Comparison of postoperative data at final follow-up

Variables^*a*^	Group NF (*N* = 40)	Group F (*N* = 18)	*P* value
Functional scores			
IKDC subjective score	69.8 ± 16.6	63.8 ± 12.1	0.176
Lysholm score	77.5 ± 17.7	76.9 ± 19.2	0.993
Tegner activity score	3.4 ± 1.7	3.2 ± 1.6	0.831
Clinical improvement beyond the MCID^*b*^			
IKDC subjective score^*c*^			
Yes/ No	26/ 14	6/ 12	0.044
Lysholm score^*c*^			
Yes/ No	25/ 15	9/ 9	0.402
Tegner activity score^*c*^			
Yes/ No	17/ 23	8/ 10	> 0.999
Knee laxity			
SSD in anterior tibial translation, 134N	1.8 ± 1.2	5.8 ± 3.5	< 0.001
Lachman test, grade^*c*^			
0/ 1/ 2/ 3	19/ 21/ 0/ 0	3/ 7/ 7/ 1	< 0.001
Pivot-shift test^*c*^			
0/ 1/ 2/ 3	18/ 22/ 0/ 0	6/ 5/ 4/ 3	< 0.001
Radiographic parameters			
Kellgren–Lawrence grade^*c*^			
0/ 1/ 2/ 3	23/ 13/ 4/ 0	2/ 4/ 11/ 1	< 0.001

*IKDC* International Knee Documentation Committee, *MCID* minimal clinically important differences, *SSD* side-to-side difference

^*a*^The values are given as the mean and standard deviation, otherwise noted separately

^*b*^Comparison between before surgery and final follow-up

^*c*^The values are given as number of patients

To analyze the independent factors influencing surgical failure after revision ACL reconstruction, multivariate logistic regression analysis was performed. Variables that exhibited statistically significant differences in previous between-group comparisons for demographic, perioperative, and intraoperative data were eligible for inclusion in the regression model. Consequently, the variables identified as statistically significant independent factors influencing surgical failure were whether patients underwent multiple revision ACL reconstruction, preoperative osteoarthritis grade, and femoral tunnel depth (Table 5). The post hoc power of the logistic regression analysis was 75.3%.

**Table 5:** Multivariate logistic regression analysis of factors affecting surgical failure after revision ACL reconstruction

Variables^*a*^	β Coefficient	Odds ratio (95% confidence interval)	*P* value
Multiple-revision surgery			
No	Reference	Reference	
Yes	3.863	47.602 (3.007–753.501)	0.006
Preoperative Kellgren-Lawrence grade			
0	Reference	Reference	
1	1.587	4.887 (0.659–36.238)	0.121
2	3.248	25.75 (2.091–317.139)	0.011
Femoral tunnel position, depth	0.203	1.225 (1.025–1.465)	0.026

*ACL* anterior cruciate ligament

^*a*^Statistically significant variable in preceding comparative analysis

## Discussion

The most principal finding of the present study is that surgical failure after revision ACL reconstruction can occur in a substantial number of cases, and various factors may influence such surgical failure. Independent factors affecting surgical failure after revision ACL reconstruction include cases corresponding to multiple revision ACL reconstruction, preoperative osteoarthritis grade, and femoral tunnel depth. The findings of this study could contribute not only to improving the outcomes of revision ACL reconstruction but also to providing valuable insights during preoperative consultations with patients.

Surgical failure following revision ACL reconstruction poses a formidable challenge for orthopedic surgeons. Managing surgical failure after revision ACL reconstruction necessitates meticulous consideration of various aspects, as is common in any surgery. Addressing factors, such as age, concurrent intra-articular pathology, graft selection, tunnel widening, and the potential need for combined osteotomy or extra-articular procedures, can pose greater complexity compared with primary surgery [1, 13, 35, 36]. Moreover, the outcomes of revision ACL reconstruction are reported to be inferior to those of primary ACL reconstruction [8–11]^)^. Hence, it is imperative to elucidate the factors influencing surgical failure after revision ACL reconstruction. While these factors are anticipated to bear similarities to those after primary ACL reconstruction, clinical studies to validate this assumption are essential. Identifying modifiable factors influencing surgical failure after revision ACL reconstruction could mitigate the risk of failure, thereby enhancing surgical outcomes. Even if the discovered factors are non-modifiable, they could still provide valuable insights during preoperative consultations. For these reasons, this study conducted an analysis of factors contributing to surgical failure after revision ACL reconstruction.

This study has unveiled that factors, such as cases corresponding to multiple revision ACL reconstruction, preoperative osteoarthritis grade, and femoral tunnel depth, may influence surgical failure after revision ACL reconstruction. While not entirely identical, the results of this study align with previous research findings. The Multicenter ACL Revision Study (MARS) group, a leading contributor to research on revision ACL reconstruction, reported that cases of multiple revision surgery and degenerative changes in the knee may be associated with poor surgical results after revision ACL reconstruction [14, 18, 19]. The degenerative changes in the knee, represented in this study through the preoperative osteoarthritis grade, may adversely affect the ACL graft by intensifying inflammatory responses within the joint [37, 38]. Remarkably, unlike preoperative radiographic osteoarthritis grade, there were no differences between groups regarding cartilage status observed during surgical procedures. However, this may be attributed to the inclusion of patients who underwent surgical restoration for cartilage lesions in the analysis, and it is also well known that radiographic osteoarthritis grade does not perfectly correlate with arthroscopic findings [39]. Furthermore, for the case of multiple revision ACL reconstruction, it is expected that degenerative changes in the knee will accelerate in the process of repeated surgical failure and be reflected in the surgical results. Indeed, a recent systematic study reported that degenerative changes in the meniscus and cartilage are more frequent in re-revision ACL reconstruction compared with primary or revision surgery [15]. Although the two factors mentioned above are not modifiable by the surgeon, such insights can offer valuable assistance during preoperative patient consultations.

Beyond non-modifiable factors (multiple revision surgery and preoperative osteoarthritis grade), it has been observed that there are factors influencing the surgical outcomes of revision ACL reconstruction that can be controlled by the surgeon, such as femoral tunnel depth. The non-anatomic placement of the femoral tunnel is recognized as one of the most common technical errors impacting surgical failure after ACL reconstruction [40, 41], and this effect is presumed to have also applied to the patients included in this study. This study reveals a significant association between a shallower femoral tunnel depth and an elevated risk of surgical failure. Notably, Byrne et al. reported an increased risk of non-traumatic ACL failure when the femoral tunnel is relatively anteriorly (shallowly) positioned [42]. Admittedly, although a significant difference in femoral tunnel depth was observed between groups in this study, this difference may not be as pronounced in practical surgical situations. Nevertheless, this finding emphasizes the importance of precise tunnel positioning during surgery. While revision surgery is inherently more challenging, surgeons should endeavor to minimize the risk of surgical failure by anatomically positioning the femoral tunnel as much as possible.

The entirety of factors that could potentially impact surgical failure following revision ACL reconstruction might not have been fully elucidated in this study. An analysis of still-debatable factors, such as graft selection [43–45], could have produced different results if evaluated with a larger sample size. However, notwithstanding these limitations, the strength of this study lies in its comprehensive analysis, encompassing intraoperative data to radiographic assessments using various imaging modalities. Information on non-modifiable factors revealed to influence surgical failure after revision ACL reconstruction, such as cases of multiple revision reconstructions and preoperative osteoarthritis grade, can be beneficial in patient selection and preoperative consultations. Concurrently, striving for more precise femoral tunnel placement, a modifiable factor during surgery, may reduce the risk of surgical failure. This study could serve as a foundational basis for formulating surgical strategies for revision ACL reconstruction.

The present study has several limitations. First, as a single-center study, the number of patients included in this research is limited owing to the relatively infrequent nature of revision ACL reconstruction compared with primary surgery. Although unavoidable, this limited sample size may have influenced the analysis results. Second, this study relies on retrospective record review, which may lead to biased assessments. Third, not all patients included in this study underwent postoperative CT and MRI evaluations; however, the frequency of each examination did not exhibit between-group differences. Fourth, patients who underwent re-revision ACL reconstruction were also included in this study. However, as previously stated, this was done to assess the impact of multiple ACL reconstructions. Nonetheless, even in the comparative analysis excluding patients subjected to multiple revision surgery, the findings remained unchanged. Finally, although this study conducted a comprehensive evaluation of all possible factors associated with the surgical outcomes, including technical errors, there might be unassessed factors that could potentially influence surgical outcomes.

## Conclusions

Surgical failure after revision ACL reconstruction can occur in a substantial number of patients, influenced by non-modifiable factors, such as cases corresponding to multiple revision surgery and preoperative osteoarthritis grade, and modifiable factors, such as femoral tunnel depth.

## Supplementary Information

Supplementary Material 1.

## Data Availability

The datasets used and/or analyzed during the current study are available from the corresponding author upon reasonable request.

## Abbreviations

ACL: Anterior cruciate ligament
MRI: Magnetic resonance imaging
AP: Anterior–posterior
IKDC: International Knee Documentation Committee
MCID: Minimal clinically important difference
CT: Computed tomography
3D: Three-dimensional
ICCs: Intraclass correlation coefficients
