# Supplementary material for: Modifiable and non-modifiable risk factors affecting surgical failure after revision ACL reconstruction: a cohort study
Source: Knee Surg Relat Res. 2024 Nov 25;36:37. doi: 10.1186/s43019-024-00243-4 (PMC11590207; doi:10.1186/s43019-024-00243-4)
Supplement: Supplementary file 1 — Supplementary Material 1. [file 43019_2024_243_MOESM1_ESM.docx]

**Supplementary Table 1** Comparison of Baseline Demographic Data Excluding Patients Corresponding to Re-revision ACL Reconstruction

| Variables*^a^* | Group NF  (N=39) | Group F  (N=11) | *P* Value |
| --- | --- | --- | --- |
| Age, year | 27.8 ± 9.3 | 28.7 ± 12.7 | 0.888 |
| Sex*^b^* |  |  |  |
| Male/ Female | 34/ 5 | 9/ 2 | 0.641 |
| Affected side*^b^* |  |  |  |
| Right/ Left | 33/ 6 | 7/ 4 | 0.197 |
| Body Mass Index, kg/m^2^ | 26.0 ± 3.7 | 24.8 ± 3.7 | 0.336 |
| Postoperative CT scan*^bc^* |  |  |  |
| Yes/ No | 36/ 3 | 10/ 1 | > 0.999 |
| Postoperative MRI*^bd^* |  |  |  |
| Yes/ No | 26/ 13 | 7/ 4 | > 0.999 |
| Pre-injury Tegner activity score | 5.3 ± 1.8 | 5.0 ± 1.5 | 0.63 |
| Follow-up duration, year | 3.2 ± 1.4 | 3.8 ± 1.4 | 0.06 |

CT, computed tomography; MRI, Magnetic Resonance Imaging; ACL, anterior cruciate ligament; NA, not applicable.

*^a^* The values are given as the mean and standard deviation, otherwise noted separately

*^b^* The values are given as number of patients.

*^c^* Images taken immediately after surgery.

*^d^* Images taken 1 year after surgery. ±

**Supplementary Table 2** Comparison of Peri-operative Data Excluding Patients Corresponding to Re-revision ACL Reconstruction

| Variables*^a^* | Group NF  (N=39) | Group F  (N=11) | *P* Value |
| --- | --- | --- | --- |
| Pre-operative |  |  |  |
| Functional scores |  |  |  |
| IKDC subjective score | 50.1 ± 15.6 | 51.5 ± 13.6 | 0.791 |
| Lysholm score | 60.8 ± 23.8 | 63.0 ± 21.9 | 0.786 |
| Tegner activity score | 1.9 ± 1.6 | 2.4 ± 1.3 | 0.174 |
| Knee laxity |  |  |  |
| SSD in anterior tibial translation, 134N | 6.4 ± 2.8 | 7.9 ± 2.6 | 0.109 |
| Lachman test, grade*^b^* |  |  |  |
| 0/ 1/ 2/ 3 | 0/ 10/ 20/ 9 | 0/ 0/ 8/ 3 | 0.151 |
| Pivot-shift test, grade*^b^* |  |  |  |
| 0/ 1/ 2/ 3 | 1/ 8/ 17/ 12 | 0/ 5/ 2/ 4 | 0.287 |
| Radiographic parameters |  |  |  |
| Kellgren-Lawrence grade*^b^* |  |  |  |
| 0/ 1/ 2 | 26/ 11/ 2 | 2/ 5/ 4 | 0.003 |
| Hip-Knee-Ankle angle, ° | 1.2 ± 3.2 | 0.0 ± 4.4 | 0.301 |
| Posterior Tibial Slope, ° | 8.3 ± 2.8 | 9.1 ± 2.7 | 0.412 |
| Lateral femoral condyle ratio, % | 65.6 ± 2.6 | 66.5 ± 4.2 | 0.426 |
| Post-operative |  |  |  |
| Femoral Tunnel Position*^c^* |  |  |  |
| Height, % | 48.5 ± 7.9 | 45.0 ± 7.7 | 0.223 |
| Depth, % | 28.7 ± 5.4 | 35.8 ± 7.8 | 0.007 |
| Tibial Tunnel Position*^c^* |  |  |  |
| Depth, % | 37.7 ± 4.1 | 40.3 ± 6.3 | 0.122 |
| Width, % | 44.8 ± 2.2 | 45.6 ± 1.3 | 0.174 |
| Femoral Intercondylar notch character*^c^* |  |  |  |
| Volume, cm^3^ | 9.7 ± 1.9 | 9.5 ± 2.3 | 0.772 |
| Distal base area, mm^2^ | 475.5 ± 73.4 | 473.5 ± 62.9 | 0.936 |
| Proximal base area, mm^2^ | 243.9 ± 41.9 | 227.9 ± 46.9 | 0.303 |
| Height, mm | 27.2 ± 2.5 | 27.2 ± 3.2 | 0.824 |

IKDC, International Knee Documentation Committee; SSD, side-to-side difference.

*^a^* The values are given as the mean and standard deviation, otherwise noted separately

*^b^* The values are given as number of patients.

*^c^* For patients who underwent postoperative CT scan (37 in Group NF and 16 in Group F)

**Supplementary Table 3** Comparison of Intra-operative Data Excluding Patients Corresponding to Re-revision ACL Reconstruction

| Variables*^a^* | Group NF  (N=39) | Group F  (N=11) | *P* Value |
| --- | --- | --- | --- |
| Graft diameter, mm*^b^* | 9.2 ± 0.7 | 9.4 ± 0.7 | 0.467 |
| Graft type |  |  |  |
| Autograft/ Allograft/ Hybrid graft | 5/ 31/ 3 | 3/ 7/ 1 | 0.49 |
| Graft type (Detailed) |  |  |  |
| Hamstring autograft/ BTB autograft/  Tibialis anterior allograft/ Achilles allograft/ Hybrid graft | 2/ 3/ 30/ 1/ 3 | 1/ 2/ 5/ 2/ 1 | 0.096 |
| Associated intra-articular lesion |  |  |  |
| Yes/ No^b^ | 22/ 17 | 9/ 2 | 0.17 |
| Medial meniscus |  |  |  |
| Functional/ Non-functional/ Repair | 21/ 5/ 13 | 5/ 3/ 3 | 0.524 |
| Lateral meniscus |  |  |  |
| Functional/ Non-functional/ Repair | 33/ 0/ 6 | 10/ 0/ 1 | > 0.999 |
| Tibiofemoral joint cartilage |  |  |  |
| Intact or low-grade lesion/ High-grade lesion/ Restoration procedure | 33/ 1/ 5 | 10/ 1/ 0 | 0.287 |
| Patellofemoral joint cartilage |  |  |  |
| Intact or low-grade lesion/ High-grade lesion/ Restoration procedure | 37/ 0/ 2 | 9/ 1/ 1 | 0.206 |
| Combined ALL Reconstruction |  |  |  |
| Yes/ No | 8/ 31 | 2/ 9 | > 0.999 |

ALL, anterolateral ligament; BTB, bone-patellar tendon-bone.

*^a^* The values are given as number of patients.

*^b^* The values are given as the mean and standard deviation.

**Supplementary Table 4** Comparison of Postoperative Data at Final Follow-Up Excluding Patients Corresponding to Re-revision ACL Reconstruction

| Variables*^a^* | Group NF  (N=39) | Group F  (N=11) | *P* Value |
| --- | --- | --- | --- |
| Functional scores |  |  |  |
| IKDC subjective score | 69.3 ± 16.5 | 68.0 ± 11.9 | 0.807 |
| Lysholm score | 77.4 ± 17.9 | 87.4 ± 8.7 | 0.125 |
| Tegner activity score | 3.3 ± 1.7 | 3.6 ± 1.5 | 0.483 |
| Clinical improvement beyond the MCID*^b^* |  |  |  |
| IKDC subjective score*^c^* |  |  |  |
| Yes/ No | 25/ 14 | 5/ 6 | 0.311 |
| Lysholm score*^c^* |  |  |  |
| Yes/ No | 24/ 15 | 9/ 2 | 0.292 |
| Tegner activity score*^c^* |  |  |  |
| Yes/ No | 16/ 23 | 6/ 5 | 0.503 |
| Knee laxity |  |  |  |
| SSD in anterior tibial translation, 134N |  |  |  |
| Lachman test, grade*^c^* |  |  |  |
| 0/ 1/ 2/ 3 | 18/ 21/ 0 | 1/ 7/ 3 | 0.003 |
| Pivot-shift test*^c^* |  |  |  |
| 0/ 1/ 2/ 3 | 17/ 22/ 0/ 0 | 5/ 4/ 1/ 1 | 0.07 |
| Radiographic parameters |  |  |  |
| Kellgren-Lawrence grade*^c^* |  |  |  |
| 0/ 1/ 2/ 3 | 22/ 13/ 4/ 0 | 0/ 2/ 8/ 1 | < 0.001 |

IKDC, International Knee Documentation Committee; MCID, minimal clinically important differences; SSD, side-to-side difference.

*^a^* The values are given as the mean and standard deviation, otherwise noted separately

*^b^* Comparison between before surgery and final follow-up.

*^c^* The values are given as number of patients.
